# Parvalbumin interneurons under stress: converging mechanisms of vulnerability

**DOI:** 10.1093/ijnp/pyag043

**Published:** 2026-08-13

**Authors:** Thamyris Santos-Silva, Lívea Dornela Godoy, Francisco S Guimarães, Felipe V Gomes

**Affiliations:** Institut de Neurosciences de La Timone, UMR 7289, Centre National de la Recherche Scientifique and Aix-Marseille Université, Marseille 13005, France; Departamento de Fisiologia, Instituto de Ciências Biológicas, Universidade Federal de Minas Gerais, Belo Horizonte, Minas Gerais, Brazil; Translational Neuropsychiatry Unit, Department of Clinical Medicine, Aarhus University, Aarhus, Denmark; Department of Pharmacology, Ribeirão Preto Medical School, University of São Paulo, Ribeirão Preto, São Paulo, Brazil; Department of Pharmacology, Ribeirão Preto Medical School, University of São Paulo, Ribeirão Preto, São Paulo, Brazil

**Keywords:** GABA, fast-spiking interneurons, perineuronal nets, early-life stress, chronic stress, pharmacological strategies

## Abstract

From early life to adulthood, stress shapes brain circuits by impacting vulnerable neuron populations, with parvalbumin-expressing interneurons (PVIs) emerging as particularly sensitive targets. These fast-spiking interneurons orchestrate inhibitory control, maintain excitatory/inhibitory balance, and regulate network oscillations, all of which are crucial for cognitive and emotional function. In addition, PVIs play a central role in regulating stress vulnerability and resilience. Several cellular and molecular mechanisms have been implicated in stress-induced PVI deficits, including disrupted developmental trajectories, redox and metabolic vulnerabilities, inflammatory and microglia-associated signaling, and epigenetic modulation. Stress also remodels perineuronal nets (PNNs), specialized extracellular matrix structures that enwrap PVIs and contribute to their stabilization, the regulation of synaptic function, and protection against oxidative stress. This review synthesizes evidence from rodent models of stress, detailing putative mechanisms through which stress alters PVIs, their associated PNNs, and their circuit-level consequences across development and brain regions, including sex-dependent effects. It further discusses pharmacological and adjuvant interventions that may mitigate stress-induced PVI dysfunction, including monoaminergic modulators, ketamine and its derivatives, as well as anti-inflammatory and antioxidant strategies. By integrating mechanistic insights with potential strategies to protect or restore PVI function, we aim to provide a framework for understanding and reducing stress-induced psychiatric outcomes.

## Summations and considerations

Parvalbumin interneurons (PVIs) are key regulators of network dynamics and exert strong inhibitory control over pyramidal neurons, with their dysfunction representing a convergent pathophysiological mechanism across several psychiatric disorders.

Stress acts as a critical environmental factor that interacts with genetic susceptibility and developmental processes to destabilize PVI circuit function, contributing to the emergence of psychiatric disorders.

Stress-induced PVI dysfunction arises from multiple mechanisms, including altered developmental trajectories, disrupted redox and metabolic homeostasis, inflammatory signaling and PVI–perineuronal net remodeling, and persistent epigenetic modifications.

Targeting mechanisms that preserve or restore parvalbumin interneurons (PVIs) function may offer new opportunities for the prevention and treatment of psychiatric disorders associated with stress exposure.

Future studies should further investigate and clarify the developmental and temporal dynamics of stress-induced alterations in PVIs that may influence vulnerability and resilience.

## Introduction

Parvalbumin-expressing GABAergic interneurons (PVIs) are specialized for rapid, high-frequency spiking activity, enabling them to generate sustained high-frequency trains of action potentials and provide temporally precise inhibitory control over their target cells, particularly excitatory pyramidal neurons.^1^ PVIs are the most abundant subtype of cortical GABAergic interneurons, accounting for approximately 30%–40% of them,^2^^,^^3^ with an average ratio of one PVI for every 8-10 pyramidal neurons.^4^ Their critical role in maintaining the excitatory/inhibitory (E/I) balance, by preventing excessive excitation and regulating spike-timing-dependent plasticity, relies on their unique electrophysiological properties.^5^

Different subtypes of PVIs are distributed throughout the brain, and their distinct connectivity patterns contribute to the coordination of network rhythms.^6^ In cortical areas, PVIs are primarily classified morphologically as basket cells, with a smaller population of axo-axonic chandelier cells. Basket cells constitute the predominant PVI subtype in the neocortex and exert powerful perisomatic inhibition onto pyramidal neurons.^7^ In contrast, chandelier cells form axo-axonic synapses that target the axon initial segment of pyramidal neurons. These input–output connectivity patterns enable PVIs to exert precise feedforward and feedback inhibitory control within microcircuits. Through these mechanisms, PVIs contribute to the synchronization of neuronal populations.^8^

PVIs can fire at frequencies exceeding 200 Hz^6^, making them the major class of fast-spiking interneurons in cortical circuits. Through their powerful inhibition, PVIs synchronize pyramidal neuron activity and support the generation of gamma oscillations, which are critical for information processing, network coordination, and synaptic plasticity.^9^^,^^10^ Consistent with these circuit-level functions, PVIs contribute to a broad range of behavioral domains and higher-order cognitive processes, including sociability, executive function, learning and memory, sensory processing, and attention.^11–15^ Their central role in coordinating network dynamics positions PVIs as a key hub in multiple physiological and pathophysiological processes, underscoring the importance of understanding the mechanisms that regulate their function and vulnerability.

Until early adulthood, PVIs are not fully mature, as they undergo a prolonged developmental trajectory that extends into adolescence in rodents,^16^^,^^17^ monkeys,^18^ and humans.^16^ As PVIs reach functional maturity, they play a key role in regulating the timing of developmental critical periods of heightened plasticity during postnatal development.^11^ This maturation process is shaped by both intracellular and extracellular signaling, including brain-derived neurotrophic factor (BDNF), hormones, and activity-dependent synaptic remodeling.^19^^,^^20^ The developmental trajectory ultimately culminates in the formation of the perineuronal nets (PNNs), a specialized extracellular matrix structure that ensheathe the soma, proximal dendrites, and initial axon segments of a large proportion of PVIs.^21–24^ PNNs regulate PVI synapse formation and function by interacting, directly or indirectly, with neurotrophic factors, receptors, guidance cues, and glia cells.^25–28^ Together, these features render PVIs and their associated PNNs highly responsive to their microenvironment and to external perturbations.^29^^,^^30^

A growing body of evidence points to PVI abnormalities in several psychiatric disorders,^31^ including major depressive disorder,^32^^,^^33^ bipolar disorder,^34^ and schizophrenia.^35^ Chronic stress is thought to be a major contributor to this vulnerability, as it represents a well-established risk factor for mood disorders, and when occurring during critical windows of brain development such as childhood and adolescence, can also increase susceptibility to schizophrenia.^36^^,^^37^ Indeed, PVIs are highly responsive to stress throughout life, and stress exposure has been consistently associated with alterations in PVIs across multiple brain regions. In animal models, stress reduces parvalbumin (PV)-positive cell numbers or density in the prefrontal cortex (PFC) and hippocampus (HIP), while increasing them in the basolateral amygdala (BLA).^38^  *Postmortem* studies in humans reveal similar patterns, with reductions in *Pvalb* mRNA expression and PV-positive cell numbers in the PFC, hippocampal subfields, and entorhinal cortex of individuals with a history of early-life adversity.^39^^,^^40^ Reductions in PV mRNA or protein, PV immunoreactivity, or PV-positive cell counts do not necessarily indicate interneuron loss.^41^^,^^42^ PV expression is developmentally regulated and activity-dependent,^43–45^ and reduced PV labeling may therefore reflect phenotypic downregulation or reduced detectability rather than cell death. Accordingly, throughout this review, we distinguish these measures from direct evidence of interneuron loss and reserve the term PVI dysfunction for studies providing functional or circuit-level evidence.

This review focuses on the molecular and cellular mechanisms through which stress affects PVI structure and function. We discuss mechanisms proposed in various animal models of stress exposure, including increased excitatory drive, impaired PVI maturation or reduced PV expression, PV-positive cell counts or density, oxidative stress/redox dysregulation, and systemic and local changes in inflammatory markers. Rather than acting through isolated pathways, stress-induced PVI dysfunction likely reflects the interaction of redox/metabolic and inflammatory mechanisms. Across developmental stages and brain regions, these mechanisms may converge to disrupt PVI maturation and function, PNN remodeling, and inhibitory control, ultimately contributing to E/I imbalance and stress-related behavioral dysfunction. We also examine how stress impacts PVI maturation, protein expression, electrophysiological properties, E/I balance, and PNN integrity. By integrating findings from studies spanning early-life to adulthood stress exposure, we highlight evidence for differential PVI plasticity across brain regions that may shape whether stress leads to vulnerability or resilience. Finally, we discuss how pharmacological interventions may protect or counteract stress-induced PVI dysfunction, providing a translational perspective on strategies aimed at mitigating vulnerability to psychiatric disorders. By integrating mechanistic insights with therapeutic perspectives, this review provides a framework for understanding and addressing stress-related psychiatric conditions.

## Overview of stress effects on PVIs throughout development

Stress-related changes in the brain arise largely from the actions of stress-system mediators on multiple cellular targets, including neurons and glial cells.^46^ These actions can be broadly divided into non-genomic and genomic effects, corresponding to rapid, short-term modulation and transcription-dependent forms of long-term plasticity.^47^ The principal mediators orchestrating these responses in the brain include glucocorticoids, corticotropin-releasing hormone (CRH), and adrenaline/noradrenaline, although numerous other signaling molecules also contribute significantly to stress-induced adaptations and deficits.^48^ With respect to PVIs, stress-induced deficits are strongly influenced by the developmental window during which stress occurs (eg, infancy, adolescence, or adulthood) and are typically studied using well-established rodent paradigms of early-life (eg, maternal separation, limited bedding), adolescence, and adulthood stress (eg, social isolation, foot shock, chronic restraint, social defeat).^49^ Because each brain region has distinct cellular and circuit identities, stress may engage different mechanisms and drive PVI-related plasticity toward different structural and functional outcomes.^50^^,^^51^ In addition, the fast-spiking phenotype of PVIs shows distinct region- and layer-specific maturation trajectories across cortical circuits.^7^ These factors may shape the magnitude, direction, and persistence of stress-induced PVI alterations.

In this section, we summarize stress-induced changes in PV expression, PV immunoreactivity, PV-positive cell counts or density, PVI structure, and PVI function across major developmental stages and across brain regions implicated in emotional regulation and cognitive processing. The cellular and molecular mechanisms underlying these effects will be discussed in greater depth in subsequent sections of this review.

### Early-life stress

**Figure 1:**
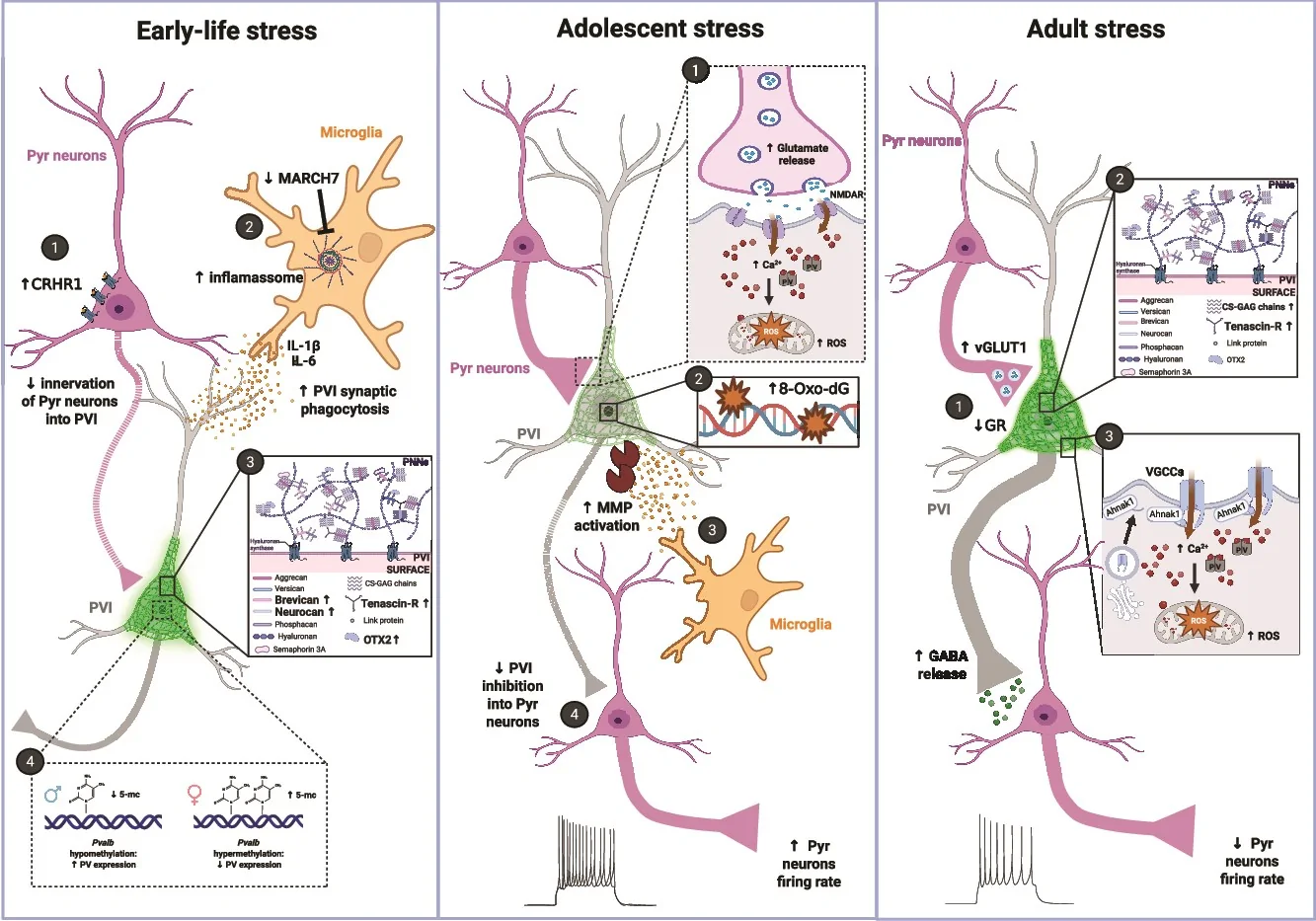
Converging mechanisms of parvalbumin interneuron (PVI) vulnerability across the lifespan. This schematic summarizes key molecular and cellular mechanisms through which stress disrupts PVI maturation and function at distinct developmental stages, ultimately converging on E/I imbalance. Early-life stress induces CRHR1-dependent alterations in pyramidal neurons, impairing excitatory innervation onto PVIs (1). It also promotes microglial activation by reducing MARCH-7, an inhibitor of the inflammasome, thereby enhancing pro-inflammatory cytokine release (IL-1β, IL-6). These inflammatory changes may contribute to PVI dysfunction and synaptic remodeling. However, direct evidence that stress induces selective microglial pruning of PVI inputs remains lacking (2). Concurrent remodeling of PNNs, characterized by increased expression of brevican, neurocan, tenascin-R, and OTX2 (3) further compromise PVI maturation and stability. Sex-dependent epigenetic changes, including changes in 5-methylcytosine intensity within PVIs (4), were associated with altered PV-positive cell counts (preliminary data). Adolescent stress primarily engages activity-dependent and oxidative mechanisms. Increased glutamatergic drive onto PVIs leads to NMDA receptor activation and Ca^2+^ overload. Due to the limited buffering capacity of PV under excessive demand, this Ca^2+^ burden may overload mitochondria, increase metabolic activity, and increase reactive oxygen species (ROS) production (1), resulting in oxidative damage (eg, 8-oxo-dG) (2). In parallel, matrix metalloproteinase (MMP) activation contributes to PNN degradation, weakening structural protection and amplifying PVI vulnerability (3). These changes reduce PVI inhibitory output and enhance pyramidal neuron excitability (4). Adult stress is characterized by dysregulation of glucocorticoid signaling and synaptic inputs. Reduced glucocorticoid receptor (GR) signaling in PVIs, together with increased excitatory drive (eg, vGLUT1 upregulation), leads to maladaptive changes in GABA release and disrupted network activity (1). Additionally, altered Ca^2+^ dynamics via voltage-gated Ca^2+^ channels (VGCCs), facilitated by increased Ahnak1 expression, further exacerbate Ca^2+^ overload and oxidative stress (2). Persistent PNN alterations, including increased CS-GAG chains and tenascin-R, further impair PVI function, contributing to network dysfunction. Although depicted as distinct pathways for clarity, these mechanisms may be biologically interconnected and converge to disrupt PVI maturation and inhibitory function, PNN remodeling, and E/I imbalance. Mechanisms enclosed by dashed-line boxes represent proposed pathways inferred from bulk tissue, non–cell-type-specific data or from a non-peer-reviewed preprint, whereas solid-line boxes indicate mechanisms directly demonstrated in PVIs.

During early life, the hypothalamic–pituitary–adrenal (HPA) axis is still undergoing maturation, and adverse experiences can profoundly shape how the developing brain responds to stress. In rodents, this developmental period encompasses the stress hyporesponsive period, during which basal glucocorticoid levels are low and physiological responses to stressors are blunted. Functional glucocorticoid signaling in the brain becomes fully operational during mid-infancy (approximately post-natal day 14 in rodents), when corticosteroid receptor expression and downstream transcriptional machinery are functional. Early-life stress can shift this developmental trajectory. Exposure to fragmented maternal care, maternal separation, or environmental adversity accelerates HPA axis maturation, leading to altered CRH signaling and adrenal responsiveness.^52^^,^^53^ This precocious activation may prematurely expose developing PVIs to elevated levels of CRH, glucocorticoids, and other stress mediators that would normally be encountered only later in postnatal development. Such mismatches can disrupt normal maturation programs. Mechanistically, early-life stress upregulates CRH receptor 1 (CRHR1) in prefrontal pyramidal neurons, altering the development of excitatory projections from pyramidal neurons onto PVIs in the PFC. This leads to reduced excitatory drive onto PVIs, decreased interneuron activity, and cognitive deficits^54^ (Figure 1). Chemogenetic and optogenetic studies indicate that restoring PVI activity or blocking CRHR1 signaling reverses these deficits, establishing a CRHR1-pyramidal neuron-PVI pathway as a mechanism underlying stress-induced dysfunction during development.

During early development, the brain undergoes pronounced plasticity associated with PVI maturation. However, findings about the impact of stressors during this period remain somewhat heterogeneous. Some reports showed increased PV immunoreactivity and PV-positive cell counts in the ventral HIP following maternal separation,^55^ whereas others detected no changes in PV or PNNs.^55^^,^^56^ In contrast, more severe adversity paradigms combining limited bedding, wire mesh exposure, and maternal separation induce anxiety- and depression-like behaviors, social deficits, and memory impairments accompanied by reduced cortical PV gene expression. Clinical evidence supports these findings. *Postmortem* studies of the human ventromedial PFC revealed protein alterations in PVIs associated with a history of childhood abuse.^40^

Early-life stress may also produce region-specific changes in the maturation trajectory of PVIs. In scarcity models, accelerated maturation of PVIs has been reported in the BLA and HIP,^57^^,^^58^ whereas reduced PV-positive cell density and altered PVI-related maturation in the orbitofrontal cortex.^59^ These findings suggest that stress may accelerate PVI maturation in some regions while delaying it in others. In the PFC and BLA, both acute and chronic stress paradigms applied during early life, including scarcity models, maternal separation, acute restraint stress, and chronic unpredictable stress, have been shown to decrease PV-positive cell counts and stability. These alterations lead to disinhibition and increased excitability of glutamatergic neurons, which may contribute to the development of anxiety-like behaviors^59^^,^^60^ and persistent maladaptive fear responses.^61^^,^^62^ The region-specific effects of early-life stress on PV- and PNN-related measures are summarized in Table 1.

**Table 1:** Early-life stress–associated PVI/PNN alterations in preclinical studies.

Stress model	Stress period	Assessment	Animal	Brain region/circuit	PVI and PNN-related outcomes	Reference
*Maternal separation*	P9, 24 h	Adulthood (P60)	Male Wistar rats	Cg1, PL, IL, CA1, CA3, DG	↓ PNN+ and PNN/PV+ density in Cg1 and IL; no change in CA1, CA3, or DG.	56
	P9, 24 h	Adulthood (P60)	Male Wistar rats	BLA and NAc	↓ PV+ density and soma size in BLA and NAc	61
	P2-14, 2 weeks	Adulthood (P74)	Male SD rats	HIP	↑ PNN components (brevican, neurocan, tenascin-R).	97
	P2-14, 2 weeks	Adulthood (P70)	Both sexes pooled C57BL/6 J mice	DG	↓ PV+ density; ↑ OTX2 in PNN-enwrapped PV+ cells.	98
	P2-P20, 3 weeks	Adolescence (P40) and adulthood (P60)	Both sexes SD rats pooled	Amygdala	↓ PV+ cell counts.	60
	P2-20, 3 weeks	Adolescence (P43)	Male SD rats	PL	↓ PV+ cell counts; ↑ GluN2A expression.	87
	P2-20, 3 weeks	Early life (P20)	Male and female SD rats	PL, IL, CA1, DG	↑ 8-Oxo-dG in PV+ cells in PL, CA1, and DG (both sexes).	116
	P2-20, 3 weeks	Adolescence (P40)	Male SD rats	PL and HIP	↓ PV+ cell counts; IL-10 prevented PV reduction and GluN2A upregulation.	125
	P2-20, 3 weeks	Adolescence (P25 and P40)	Male and female SD rats	PL	P25: ↓ PV protein and PV+ counts in females. P40: ↓ PV protein and PV+ counts in males; prevented by NS-398.	127, 128
*Limited bedding*	P2-9, 1 week	Adolescence (P35-42)	Male C57BL/6 mice	Medial PFC; pyramidal neuron-PVI circuit	↓ PVI activity, excitatory drive, and intrinsic excitability.	54
	P4-11, 1 week	Early life (P16, 21) and adolescence (P28 and 50)	Male C57BL/6 N mice	BLA	↑ PV+ density at P16 only; non-significant increase at later time points.	58
	P4-11, 1 week	Adulthood (P65-66)	Both sexes pooled C57BL/6 N mice	Orbitofrontal cortex	Female-specific ↓ PV expression and PV+ density.	59
*Maternal separation + limited bedding*	P2-14, 2 weeks	Adolescence (P56)	Both sexes pooled C57BL/6 mice	PFC	↓ PV+ density.	57
	P2-20, 3 weeks	Adolescence (P42-49)	Male C57BL/6 mice	Ventral HIP	↑ PV and GAD immunoreactivity.	55

Abbreviations: 8-oxo-dG, 8-oxo-2′-deoxyguanosine; BLA, basolateral amygdala; CA1, cornu ammonis 1; CA3, cornu ammonis 3; Cg1, cingulate cortex area 1; COX-2, cyclooxygenase-2; DG, dentate gyrus; GAD, glutamate decarboxylase; GluN2A, N-methyl-D-aspartate receptor subunit 2A; HIP, hippocampus; IL, infralimbic PFC; IL-10, interleukin-10; NAc, nucleus accumbens; NS-398, selective cyclooxygenase-2 inhibitor; P, postnatal day; PFC, prefrontal cortex; PL, prelimbic PFC; SD, Sprague–Dawley.

### Adolescent stress

Adolescence represents a particularly sensitive developmental window, as many psychiatric disorders emerge during this period, with a peak/median age at onset of 14.5/18 years across all mental disorders.^63^ Stress exposure during adolescence can have a profound impact on brain development and represents a major risk factor for the emergence of psychiatric conditions.

During this developmental stage, the brain undergoes substantial refinement of prefrontal circuits and their connections to other regions. These changes are accompanied by a marked shift in the E/I balance driven in part by a surge in PV-positive cell counts and PVI activity.^64^^,^^65^ Supporting the importance of this process, chemogenetic inhibition of PVIs during the juvenile and adolescent periods in mice leads to persistent deficits in adult PFC circuit connectivity, network function, and behavioral flexibility, whereas reversible suppression in adulthood produces no lasting effects.^66^ This delicate maturational process is highly vulnerable to stress. For instance, social isolation during adolescence prevents the normal developmental increase in PVI activity. As a consequence, stressed adolescents exhibit reduced cortical PVI function due to both decreased excitatory drive and reduced intrinsic excitability.^67^ Importantly, these effects may not appear immediately after stress exposure but instead emerge gradually as disrupted developmental trajectories unfold across adolescence, ultimately producing long-lasting deficits that persist into adulthood.^68^

The resulting reduction in inhibitory tone caused by PVI dysfunction represents a key mechanism underlying stress-related behavioral deficits. For example, in models of adolescent learned helplessness, reduced activation of inhibitory neurons in the medial PFC has been observed, and direct suppression of PVI activity is sufficient to enhance helpless behavior.^69^ Similarly, repeated footshock stress during adolescence disrupts PVI function in the PFC and leads to persistent behavioral alterations, including anxiety-like behaviors, social deficits, and cognitive impairments.^70^ The main effects of adolescent stress on PVI-related outcomes are summarized in Table 2.

**Table 2:** Adolescent stress–associated PVI/PNN alterations in preclinical studies.

Stress model	Stress period	Assessment	Animal	Brain region/circuit	PVI and PNN-related outcomes	Reference
*Post-weaning social isolation*	P21-35, 2 weeks	Adulthood (P56-63)	Male C57Bl/6 mice	Dorsomedial PFC	↓ PVI activity, excitatory drive, and intrinsic excitability.	67
	P28-63, 5 weeks	Adulthood (P63)	Male C57Bl/6 mice	Medial PFC	↓ PV+ cell counts.	151
	P21-56, 5 weeks	Adolescence (P49 and 56)	Male Wistar rats	PFC and NAc	↓ PV protein in PFC and NAc; reversed by apocynin.	183, 182
	P21-56, 11 weeks	Adolescence (P56)	Male C57BL/6 N mice	Somatosensory, visual, motor, prefrontal, temporal cortex and HIP (CA1, CA3, DG)	↓ PV+ density in DG; ↓ PNN marker in PL; ↓ PNN/PV ratio in visual, prefrontal, and hippocampal regions.	103
	P23-100, 11 weeks	Adulthood (P100)	Female SD rats	CA1, CA2/3, DG	↓ PV+ cell counts in CA2/3 and DG.	104
*Repeated footshock + restraint*	P31-40, 10 days	Adulthood (P74)	Male and female C57BL6 mice	PL	Males: ↓ PV+, PNN+, and PV/PNN+ counts. Females: outcome differed by vulnerability phenotype.	70
	P31-40, 10 days	Adolescence (P41 and 51) and adulthood (P75)	Male SD rats	Ventral HIP	↓ PV+ (P51, P75), PNN+ (P41), and PV/PNN+ counts (P51, P75).	101
	P31-40, 10 days	Adolescence (P51)	Male SD rats	Ventral HIP	↓ PV+, PNN+, and PV/PNN+ counts; ↑ 8-Oxo-dG in PV+ cells; altered redox/mitochondrial measures.	117
	P31-40, 10 days	Adolescence (P51 and 65);	Male SD rats	Ventral HIP	↓ PV+ and PNN+ counts; ↑ 8-Oxo-dG in PV+ cells; attenuated by post-stress NAC.	182
*CUMS*	P28-63, 5 weeks	Adolescence (P43) and Adulthood (P63)	Male Wistar rats	Medial/orbitofrontal PFC	Altered PNN distribution/coverage; PV outcome not consistently reported.	102
*Learned helplessness*	P35-42, 1 week	Adolescence	Male C57BL/6 N mice	Medial PFC	↓ recruitment/activation of inhibitory neurons, including PVIs.	69

Abbreviations: 8-oxo-dG, 8-oxo-2′-deoxyguanosine; AC, auditory cortex; CA1, cornu ammonis 1; CA2/3, cornu ammonis 2/3; CUMS, chronic unpredictable mild stress; DG, dentate gyrus; HIP, hippocampus; NAc, Nucleus accumbens; NAC, N-acetylcysteine; P, postnatal day; PFC, prefrontal cortex; PL, prelimbic PFC; SD, Sprague–Dawley

### Adult stress

In the mature brain, the dynamic balance between mineralocorticoid receptor (MR) and glucocorticoid receptor (GR) signaling regulates cellular and circuit responses to hormonal stress.^47^ While GR is notorious for its genomic mechanisms, it also has non-genomic effects on neuron populations, together with MR. In the HIP, PVIs and somatostatin (SST) interneurons represent putative direct targets of glucocorticoids through MR- and GR-mediated signaling pathways. However, heterogeneous patterns of GR expression suggest that stress responses may be both cell-type and region-specific. Whereas SST interneurons are ubiquitously GR-positive, the more numerous PVIs exhibit more restricted GR expression, implying functional differences in how these interneuron populations may regulate hippocampal circuits under stress.^71^

Glucocorticoids can also alter PVI function indirectly through non-genomic mechanisms. For example, glucocorticoids promote nitric oxide (NO) release from GR-expressing pyramidal neurons. Retrograde NO signaling can enhance GABA release and increase PVI activity, yet it may simultaneously reduce the number of PV-positive cell counts and disrupt rhythmic synaptic inhibition generated by these interneurons.^72^ After chronic stress, GR colocalization is significantly reduced specifically in PVIs of the infralimbic PFC. This change is accompanied by increased inhibitory neurotransmission and a greater number of GABA release sites.^72^ These findings suggest that chronic stress in adulthood creates a state characterized by both increased excitatory drive onto PVIs together with enhanced inhibitory output from these interneurons onto principal neurons in the PFC. Such dysregulation may disrupt PVI networks, impair rhythmic oscillatory activity, and contribute to the cognitive deficits commonly observed in stress-related psychiatric disorders.^73^

Genomic mechanisms may also contribute to stress-induced PVI deficits in adulthood. In the PFC, four weeks of chronic stress increase the number of PV-positive cells expressing the activity-dependent markers cFos and delta-FosB, which reflect gene transcription.^74^ Electrophysiological recordings further indicate hyperexcitability of these prefrontal PVIs, a phenotype associated with reduced overall PFC activity.^75^ This PVI hyperexcitability may result from an increased density of glutamatergic VGLUT1-positive terminals onto PVIs.^76^

Chronic stress also increased the number of Kv3.1/PV double-positive cells in the prelimbic cortex of females, whereas no significant change was observed in males.^77^ Kv3.1 channels are essential for the fast-spiking phenotype of PVIs because they enable rapid action potential repolarization and sustained high-frequency firing.^78^ Consequently, alterations in Kv3.1 expression may impair PVI function and disrupt network dynamics.

Disruption of inhibitory control may also extend to the amygdala. Chronic stress alters GABAergic signaling within the amygdaloid complex, producing hyperactivity of amygdala circuits,^79^ in contrast to the hypoactivity typically observed in the PFC.^80^ Adult stress triggers amygdala hyperexcitability and fear dysregulation by reducing BLA PV-positive cell counts accompanied by impaired PVI-mediated inhibition and increased glutamatergic excitability.^62^ Under physiological conditions, PVIs provide tonic GABA_B_ receptor-mediated inhibition to principal neurons in the lateral amygdala, a process facilitated by GluK1-containing kainate receptors. Chronic stress selectively downregulates GluK1 in PVIs, diminishing this inhibitory tone and disinhibiting lateral amygdala circuits. The resulting hyperexcitability shifts excitatory drive within the centrolateral amygdala, providing a mechanism linking chronic stress to increased anxiety and fear generalization.^81^

Taken together, adult stress does not produce a uniform increase or decrease in PVI-related measures. In the PFC, increased PVI recruitment, excitability, or inhibitory output may coexist with reduced PV labeling or disrupted rhythmic inhibition, reflecting differences in stress paradigm, duration, time of assessment, and the endpoint measured.^72^^,^^74–76^ Increased activity may therefore represent maladaptive recruitment or a compensatory response rather than enhanced inhibitory control. In other circuits, stress affects distinct molecular and synaptic properties of PVIs, including mechanisms supporting fast-spiking activity and PVI-mediated inhibitory tone. Thus, apparently divergent findings are best interpreted as context-dependent forms of PVI dysregulation rather than contradictory effects on a homogeneous interneuron population (Table 3).

**Table 3:** Adult stress–associated PVI/PNN alterations in preclinical studies.

Stress model	Stress period	Assessment	Animal	Brain region/circuit	PVI and PNN-related outcomes	Reference
*CUMS*	P63-77, 2 weeks	Adulthood (P74)	Male SD rats	IL	↓ GR/PV colocalization; altered PV-immunoreactive cell counts.	72
	P63-119, 8 weeks	Adulthood (P92 and P120)	Male and female C57BL/6 J mice	mPFC	↑ PV/c-Fos and PV/ΔFosB labeling; ↑ PVI excitability, sex- and time-dependent.	75
	P63-119, 8 weeks	Adulthood (P92 and P120)	Male and female BALB/c mice	PFC	Females: ↑ PV protein, PV+/pERK1/2+ cells, and VGLUT1+ inputs onto PVIs. Males: ↑ VGLUT1+ inputs onto PVIs.	76
	P56-91, 5 weeks	Adulthood (P92)	Male and female C56Bl/6 J mice	PL and IL	Females: ↑ PV+ counts in PL/IL and Kv3.1/PV+ cells in PL. Males: no changes	143
	>P60; 9 weeks)	Adulthood (>P60, 24 h after stress)	Male Wistar rats	IL	Vulnerable: ↓ PV+ density and GABAergic transmission; resilient: unchanged.	138
	>P60, 8 weeks)	Adulthood (>P60, 24 h after stress)	Male Wistar rats	DG	No change in DG PV+ density across groups.	140
*Acute stress or CUMS*	P63-65 or P63-77, 2 weeks	Adulthood (P67 and P76)	Male C57BL/6 N mice	BLA/lateral amygdala	↓ PV+ counts; BLA PVI activation reversed stress-induced glutamatergic hyperexcitability.	62
*Prepubertal OVX + adult CUMS*	>P60, 4 weeks	Adulthood (P80)	Female C57Bl6/J mice	Dorsal HIP	No overall change in PL PV expression or PV+ cell number; OVX × E2 × stress interaction in PV/FosB colocalization.	149
*Chronic restraint stress*	P84-93, 10 days	Adulthood (P94)	Male C57BL/6 N mice	Lateral amygdala	No change in *Pvalb*+ neuron density; ↓ Grik1 expression in PVIs and reduced PVI-mediated tonic GABAB inhibition.	82
	P91-100, 10 days	Adulthood (P101)	Male SD rats	mPFC, BLA, dorsal and ventral HIP	↑ PV+ counts in mPFC, BLA, and reticular thalamic nucleus; PNN density ↑ in mPFC/reticular thalamic nucleus and ↓ in BLA; PV/PNN+ counts unchanged; ↑ PSA-NCAM in HIP.	105
	>P60, 1 week	Adulthood (>P60, 24 h after stress)	Both sexes C57BL/6 J mice pooled	Barrel cortex	PV+ density unchanged; ↓ PV/c-Fos + cells and PVI intrinsic excitability.	152
*Social defeat stress*	P112-116, 5 days	Adulthood (P136, P146 and 176)	Male Long-Evans rats	CA1	PV+ density unchanged; PV/PNN+ counts ↓ early, recovered, then ↑ at late assessment.	106
	P77-87, 10 days	Adulthood (P89)	Male C57BL/6 mice	Ventral DG	Susceptible: ↑ putative PVI firing and Ahnak expression in PVIs. Resilient: ↓ Ahnak expression. PV-specific Ahnak deletion reduced PVI firing and promoted resilience; differential oxidative-phosphorylation signatures.	118
	>P60, 5 or 10 days	Adulthood (>P60, 24 h after stress)	Male C57BL/6 J mice	Ipsilateral MG to PVI-A1 cortex	Resilient: ↑ MG → A1 synapses onto A1 PVIs via BDNF–TrkB signaling.	153
*Escapable or inescapable tailshock*	>P60, single session	Adulthood (>P60, single session)	Male SD rats	PL, IL and lateral amygdala	PL/IL: ↑ c-Fos intensity in PV+ and PV/PNN+ cells after escapable and inescapable stress. Lateral amygdala: increase only after escapable stress; ↑ c-Fos + PV/PNN+ cell number in IL only after escapable stress.	139

Abbreviations: A1, primary auditory cortex; Ahnak, neuroblast differentiation-associated protein AHNAK; BDNF, brain-derived neurotrophic factor; BLA, basolateral amygdala; CA1, cornu ammonis 1; c-Fos, cellular Fos; CUMS, chronic unpredictable mild stress; DG, dentate gyrus; E2, 17β-estradiol; GABA, gamma-aminobutyric acid; GR, glucocorticoid receptor; HIP, hippocampus; IL, infralimbic PFC; Kv3.1, voltage-gated potassium channel subfamily KQT member 3.1; MG, medial geniculate body; mPFC, medial prefrontal cortex; OVX, ovariectomy; P, postnatal day; PFC, prefrontal cortex; pERK1/2, phosphorylated extracellular signal-regulated kinases 1/2; PL, prelimbic PFC; PNN, perineuronal net; PSA-NCAM, polysialylated neural cell adhesion molecule; PV, parvalbumin; *Pvalb*, parvalbumin gene; PVI, parvalbumin interneuron; SD, Sprague–Dawley; TrkB, tropomyosin receptor kinase B; VGLUT1, vesicular glutamate transporter 1; ΔFosB, truncated FosB isoform.

## Mechanisms underlying PVI vulnerability to stress

For organizational purposes, the following sections discuss developmental, redox/metabolic, and inflammatory mechanisms separately. However, these processes are biologically interconnected and may reinforce one another in shaping stress-induced PVI dysfunction.

### Stress impact on the developmental trajectory of PVIs

Critical periods of plasticity are temporal windows during neurodevelopment in which neuronal circuits are highly sensitive to experience, allowing functional and structural refinement. The concept emerged from classic studies of the visual cortex by Hubel and Wiesel in 1962.^82^ Decades later, evidence revealed that the onset of these plastic periods is tightly regulated by the maturation of PVIs and the establishment of GABAergic inhibitory tone.^83^

In rodents, critical periods typically extend from early postnatal stages to late adolescence, paralleling the maturation of PVIs as they acquire their fast-spiking phenotype and establish precise inhibitory control over excitatory networks.^17^^,^^84^^,^^85^ The proper timing of critical period opening and closure, therefore, depends on the maturation and activity of PVIs.^83^ Perturbations that alter PVI function during critical periods can have lasting consequences for circuit refinement. A key participant in this process is the glutamate N-Methyl-D-Aspartate (NMDA) receptor, which has been proposed to play a unique role in PVI development.^86^ Early-life stress can disrupt PVI maturation, as maternal separation leads to overexpression of the GluN2A subunit of the NMDA receptor in the adolescent prelimbic PFC, which co-occurs with reduction of PV-positive cell counts.^87^ Administration of TAT2A, a GluN2A-specific blocking peptide, during early adolescence prevents PV-positive cell reduction in rats exposed to early-life stress and reduces anxiety-like behaviors.^87^

Molecular regulators of PVI development, such as BDNF, are also highly sensitive to stress during this period.^88^^,^^89^ BDNF plays a central role in PVI maturation and critical period plasticity by promoting PVI differentiation, survival, and synaptic integration via activation of its high-affinity receptor TrkB, which initiates intracellular signaling cascades essential for neuronal plasticity and circuit stabilization.^19^^,^^20^^,^^89^ Stress may disrupt these processes by downregulating BDNF signaling and impairing its downstream effects. Consistent with this idea, pharmacological blockade of TrkB receptors at postnatal day 7 recapitulates key features of the maternal separation model, including a reduction in PV-positive cell counts in the medial PFC during adolescence, which normalize by adulthood.^90^ However, the mechanistic link between stress exposure, BDNF–TrkB signaling, and PVI maturation remains to be fully elucidated.

Beyond molecular regulation, PVI maturation is closely associated with the progressive expression of PNNs. PNNs are composed mainly of chondroitin sulfate proteoglycans (CSPGs; eg, aggrecan, neurocan, brevican, versican, phosphacan), hyaluronan, tenascin, and link proteins.^91^ Their accumulation increases gradually from the juvenile period through adolescence, marking the closure of the critical period for PVI circuit plasticity.^92^ Notably, the developmental trajectory of PNNs differs across cortical regions. In the anterior cingulate cortex, juvenile and adolescent mice exhibit similar PNN coverage of PVIs,^27^ whereas in the prelimbic and infralimbic PFC, juveniles show fewer PNNs than older animals,^21^ indicating a delayed maturation. In contrast, in the BLA, overall PNN density increases with age while the subset surrounding PVIs remains stable from juvenile to adult stages.^21^ While PNNs in the medial PFC may directly stabilize PVIs and facilitate their functional maturation, in the BLA they may support broader circuit-level adaptations.^21^ By adulthood, both regions display a mature PNN landscape, with approximately 80% of PVIs enwrapped by PNNs.^93^ Humans show a similar trajectory, as PNN density in the dorsolateral PFC gradually increases postnatally, particularly around puberty.^94^ This regional and temporal pattern of PNN maturation defines the window of PVI plasticity^95^ and provides the structural substrate underlying age-dependent vulnerability to environmental stressors.^96^

Given these developmental processes, stress exposure during the PVI maturation window represents a potent modulator of neuroplasticity. Indeed, individuals exposed to childhood abuse exhibits a 3-fold increase in unmyelinated PVIs that are, nevertheless, surrounded by PNNs, whereas healthy controls exhibit a strong correlation between PVI myelination and PNN coverage.^40^ These findings suggest disruption of the normal maturation program of these cells, in which the expected coupling between myelination and PNN formation becomes uncoupled by early-life stress.

In animal models, early maternal deprivation increases hippocampal levels of core PNN components, including brevican, neurocan, and tenascin-R,^97^ accompanied by increased accumulation of homeoprotein OTX2 in PVIs surrounded by PNN^98^ (Figure 1). OTX2, which is internalized by PVIs, promotes PNN assembly and establishes a positive feedback loop that stabilizes inhibitory circuits while restricting plasticity.^99^^,^^100^ Dysregulation of this pathway by early-life stress may therefore prematurely close critical periods, contributing to long-lasting behavioral deficits.

Our group has characterized stage-dependent effects of stress on PVIs and PNNs. Repeated stress during adolescence leads to persistent reductions in PV-positive cell counts and PNN enwrapment in both the PFC and ventral HIP.^70^^,^^101^ Specifically, in the ventral HIP, these changes were accompanied by increased pyramidal neuron firing, whereas identical stress in adulthood produces only transient depressive-like changes without altering PVI or PNN markers.^101^ Enzymatic degradation of PNNs in the adult ventral HIP prior to stress exposure reproduces an adolescent-like phenotype of stress vulnerability, with reduced PV/PNN co-labeled cell counts, hyperdopaminergic activity, and behavioral deficits.^96^ These findings highlight the protective role of mature PNNs against stress-induced circuit dysfunction. Consistent with these findings, other stress models, including early adolescent isolation and chronic mild stress, also reduce the PNN/PV ratio in hippocampal and cortical regions, coinciding with impairments in social recognition and cognitive flexibility.^102–104^

Although most studies have focused on developmental effects, stress exposure in adulthood can also modify PNNs, often in the opposite direction. For example, chronic restraint stress in adult rats increases PSA-NCAM, a polysialylated form of the neural cell adhesion molecule and PNN component, in the hippocampal CA1.^105^ This change is accompanied by increased PV-positive cell counts in the medial PFC and BLA, as well as enhanced PNN density in the medial PFC.^105^ Similarly, in the social defeat stress model, cognitive impairments coincide with dysregulation of PVIs and PNNs in the HIP.^106^ Shortly after social defeat, PNN/PV-positive cell numbers are reduced. By 2-4 weeks post-stress, PNN/PV-positive co-labeled cells recover, whereas at 8 weeks their number increases, reflecting a sustained depressive-like state. PV-positive cell density remains unchanged, suggesting a specific regulation of PNNs by stress. Changes in PNN components such as CSPGs and the link proteins Tenascin-R and Hapln1 follow a similar temporal pattern (Figure 1). They decreased shortly after stress, transiently recovered at 2 weeks, and increased at later time points.^106^

Together, these findings highlight that PVI vulnerability to environmental perturbations is age-dependent across multiple brain regions and reinforce the concept of a sensitive period for circuit maturation. Also, PNN remodeling represents a substrate mediating both the short- and long-term effects of stress. These processes are also highly sensitive to metabolic and immune challenges. The following sections will explore how redox dysregulation and inflammatory signaling interfere with stress-induced abnormalities in PVI function and PNN composition across the lifespan.

### Redox and metabolic vulnerabilities of PVIs under stress

The fast-spiking phenotype of PVIs imposes substantial metabolic demands, rendering them particularly vulnerable to oxidative stress and metabolic dysregulation.^27^ PVIs exhibit intense labeling for cytochrome c, a marker of mitochondrial abundance and neuronal activity, whereas other interneuron subtypes, including calbindin-, SST-, and cholecystokinin-expressing interneurons, show heterogeneous cytochrome c levels, and calretinin-positive cells rarely display a strong signal.^107^ Consistently, cortical mRNAs associated with metabolic and mitochondrial pathways, including genes involved in the cytochrome c oxidase assembly, are substantially enriched in PVIs, highlighting their distinctive metabolic signature.^108^ Complementarily, conditional knockout mice lacking the cytochrome c oxidase assembly factor Cox10 specifically in PVIs display progressive loss of cytochrome c oxidase, impaired prefrontal high-frequency firing, increased excitatory and inhibitory inputs onto PVIs, and disrupted gamma oscillations in the PFC and HIP.^109^

A high density of mitochondria supports the large amounts of ATP required to sustain the fast and rhythmic activity of PVIs.^110^ However, this intense oxidative metabolism also makes mitochondria both a source and a target of stress-induced PVI damage. Excessive excitatory drive, as occurs under stressful conditions, increases glutamatergic input and Ca^2+^ influx through NMDA and AMPA receptors. Under physiological conditions, PV protein buffers intracellular Ca^2+^, while remaining physiological Ca^2+^ levels stimulate mitochondrial ATP production. However, under chronic or intense activation, mitochondrial Ca^2+^ accumulates to toxic levels, disrupting the mitochondrial membrane potential and impairing electron transport chain activity.^111^ This process leads to ATP depletion and increased production of reactive oxygen species, triggering a redox imbalance that is particularly harmful to PVIs due to their reliance on oxidative metabolism and relatively limited antioxidant reserves^112^ (Figure 1).

Pioneering work by Dr Kim Q. Do and colleagues demonstrated that deficits in glutathione, the major intracellular antioxidant, impair PVI maturation and function, leading to disturbed E/I balance and behavioral abnormalities.^112–114^ Indeed, the availability of cysteine, the rate-limiting amino acid in glutathione synthesis, is tightly linked to PVI activity and redox homeostasis.^115^ Notably, other interneuron subtypes, such as calretinin- and calbindin-expressing cells, appear less sensitive to oxidative stress, underscoring the unique vulnerability of PVIs due to their metabolic and electrophysiological profiles.^27^

Early-life stress can compromise mitochondrial or antioxidant function, potentially leading to persistent oxidative stress and long-lasting reduction in PV-positive cell counts in the PFC. For instance, maternal separation increases the accumulation of the oxidative DNA damage marker 8-OxodG specifically in PVIs of the medial PFC as well as in the CA1 and dentate gyrus of HIP.^116^ Our studies in rats exposed to stress during adolescence similarly revealed increased co-localization of 8-OxodG with PV-positive cells in the ventral HIP^117^ (Figure 1). We also observed increased levels of oxidized glutathione and hydrogen peroxide, reflecting sustained oxidative pressure, alongside upregulation of antioxidant defenses, likely representing a compensatory response. In addition, mitochondrial respiration was transiently elevated immediately after stress but later exhibited reduced oxidative phosphorylation capacity.^117^ Bulk RNA-seq analyses further supported this mechanism, revealing enrichment of redox-related pathways, including negative regulation of oxidative-stress–induced neuron death and glutathione transferase activity, and stress-related apoptotic pathways. SynGO analysis additionally identified significant changes in genes involved in synaptic vesicle cycle regulation, suggesting presynaptic dysfunction.^117^ Importantly, cell-type mapping using HuBMAP showed enrichment for the “Ing L5–6 PVALB MEPE—BRAIN” signature, indicating that several differentially expressed genes are preferentially expressed in PVIs. Together, these findings point to convergent oxidative, metabolic, and synaptic alterations that may underline PVI deficits and E/I imbalance in the ventral HIP of adolescent-stressed animals.

Supporting this view, studies using the chronic social defeat stress model in adulthood showed stress-specific transcriptional remodeling in PVIs of the ventral HIP.^118^ Differential gene expression analyses revealed that transcripts associated with mitochondrial oxidative phosphorylation and EIF2-dependent protein synthesis are upregulated in stress mice. Mechanistically, the scaffold protein Ahnak, which is highly expressed in PVIs, modulates L-type voltage-gated Ca^2+^ channels (VGCCs), regulating Ca^2+^ influx and downstream mitochondrial activity, ATP production, and Ca^2+^-dependent gene expression. In stressed mice, elevated Ahnak expression enhances VGCC trafficking and Ca^2+^ signaling, potentially driving mitochondrial hyperactivity and metabolic stress (Figure 1). This Ahnak–VGCC–mitochondria axis thus provides a molecular link between Ca^2+^ signaling, oxidative metabolism, and behavioral outcomes under chronic stress exposure. Complementary evidence from the chronic mild stress paradigm in rats further shows a selective decrease in PV expression in the dorsal HIP, which correlates with modulation of the antioxidant master regulator NRF2 and its inhibitory partner, the chaperone protein KEAP1.^119^

Human studies provide indirect but convergent evidence indicating that childhood trauma is associated with systemic oxidative stress, mitochondrial dysregulation, and reduced hippocampal volume in early psychosis.^120^ Although direct measures of PVI redox state or mitochondrial function in humans are currently lacking, these findings suggest a synergistic mechanism whereby early-life stress may impair PVI metabolism and compromise long-term circuit stability.

### Inflammatory signaling, microglial changes, and PVI–PNN remodeling under stress

During an early postnatal window, microglial depletion increases the density of PVI inhibitory synapses onto excitatory neurons without altering PV-positive cell number, indicating that microglia contribute to the maturation or refinement of PVI-mediated inhibition after initial synapse assembly.^121^ Physical contacts between microglia and PVIs have also been described in hippocampal CA1, although this observation provides anatomical evidence of microglia–PVI proximity rather than evidence for a stress-induced mechanis.^122^ Together, these studies establish that microglia can influence PVI inhibitory circuits, but do not demonstrate that stress recruits the same mechanism.

Direct evidence for microglia-dependent synaptic remodeling is currently stronger for excitatory than for PVI-related synapses. Chronic corticosterone exposure induces layer-specific complement activation, increased microglial lysosomal activity, and microglial engulfment of VGLUT2-positive synaptic material in layer 2/3 of the medial PFC, accompanied by a reduction in VGLUT2/PSD95-positive synapses.^123^ These findings indicate stress-induced remodeling of excitatory, likely thalamocortical, synapses. Because the postsynaptic identity of the affected synapses was not determined, it remains possible that a subset of these inputs targets PVIs. However, this was not directly tested. In contrast, VGAT/gephyrin-positive inhibitory synapse density was unchanged. Thus, this study does not indicate selective microglial pruning of PVI-mediated synapses, but it raises the possibility that stress-induced microglial remodeling of excitatory afferents could indirectly alter PVI recruitment and circuit function. Adolescent stress can also lead to alterations in microglial measures and dendritic spine density in prefrontal and hippocampal regions. In the Nrg1-deficiency model, however, the effects of stress on dendritic spines were modified in a region-specific manner without corresponding changes in stress-induced microglial activation or PV-positive cell counts.^124^

Stress-related cytokine alterations represent a potential inflammatory pathway through which immune signaling may influence PVI function. However, the cellular source of these mediators and their direct actions on PVIs are not established. Maternal separation induces long-lasting inflammatory alterations, and central administration of the anti-inflammatory cytokine IL-10 during pre-adolescence prevents maternal separation-induced reduction in PV-positive cell counts.^125^ Neuroinflammation can promote excessive glutamatergic NMDA receptor activation, to which PVIs are particularly sensitive. Notably, IL-10 treatment also prevents maternal separation-induced upregulation of the NMDA receptor subunit GluN2A,^125^ suggesting that altered glutamatergic signaling may contribute to inflammation-associated changes in PVI-related measures in the PFC. Moreover, higher circulating IL-1β and IL-6 predict lower PV expression in maternally separated animals, linking peripheral inflammation to PVI vulnerability.^125^ Furthermore, reduction in the cannabinoid receptor 2 (CB2) and its associated regulatory enzyme MARCH7 leads to increased NLRP3, a critical factor in inflammasome activity, in the prelimbic PFC of maternally separated adolescent females.^126^ Triple-label microscopy suggested that CB2 receptor and MARCH7 signals were present in microglia closely apposed to PV-positive cells.^126^ Early-life stress also increases cyclooxygenase-2 (COX-2) expression in the PFC, concurrent with PV-positive cell reduction and working memory deficits.^127^ However, these mechanisms are sex- and age-dependent. In juvenile females, maternal separation induces early PV-positive cell reduction and social interaction deficits without detectable changes in COX-2 levels. In males, by contrast, reductions in PV-positive cell number emerge later during adolescence, suggesting that COX-2–mediated changes may contribute selectively to male vulnerability.^128^

Similar patterns are observed following other early-life neurodevelopmental insults. Neonatal NMDA receptor blockade combined with post-weaning social isolation selectively reduces PV immunoreactivity and PV-positive cell density in cortical regions, whereas SST- and calbindin-expressing interneurons remain largely unchanged.^129^ Although overall expression and density of ionized Ca^2+^-binding adaptor molecule 1 (Iba-1), a microglia marker, are not altered, morphological analyses reveal increased early-stage microglial activation and a trend toward fewer resting microglia. This is accompanied by elevated IL-6 levels in the PFC, indicating a localized pro-inflammatory response.^129^ Moreover, combined exposure to stress and inflammatory insults further exacerbates PVI vulnerability. Prenatal immune activation induced by poly(I:C) administration in pregnant mice sensitizes hippocampal PVIs to subsequent peripubertal stress, as only the combination of these insults reduced PV-positive cell counts in the ventral HIP.^130^ Similarly, combined lipopolysaccharide (LPS) administration and chronic stress in mice induce microglial activation, elevate pro-inflammatory cytokines such as IL-1β and IL-6, reduce the anti-inflammatory cytokine IL-10, and lead to reduced PV-positive cell counts and impaired theta and gamma oscillations during cognitive tasks.^131^ Together, these findings suggest that stress- and inflammation-induced alterations in PVIs contribute to disrupted cortical network dynamics and behavioral deficits associated with stress-related disorders.

### Potential epigenetic contributions to stress-related PVI alterations

Early-life adversity can induce persistent changes in DNA methylation and chromatin regulation of stress-related genes, including the GR and BDNF.^132^^,^^133^ However, most of this evidence derives from bulk tissue or analyses that do not specifically identify PVIs. These findings support a broad role for epigenetic regulation in the long-term effects of stress, but do not establish that stress directly modifies the epigenetic landscape of PVIs. Preliminary findings indicate sex- and age-dependent changes in 5-methylcytosine intensity within PV-positive cells following maternal separation.^134^ However, no changes were detected at the two analysed CpG sites within the *Pvalb* promoter, and the genomic targets underlying these associations remain unknown. More direct links to PVI-related regulation have been reported in a neurodevelopmental two-hit model combining MAM-E17 exposure with adolescent social isolation.^135^ In this model, PV mRNA levels decrease in late adolescence and are accompanied by reduced total and *Pvalb*-bound H3K4me3 levels, whereas PV protein levels paradoxically increase. In adulthood, PV protein levels decrease without affecting mRNA or H3K4me3 levels. Interestingly, this two-hit paradigm does not alter PV-positive cell numbers at any age. These findings suggest that adolescent social isolation acts as a stressor that accelerates epigenetic impairments of PV expression in the MAM-E17 model, in which animals exhibit increased stress responsivity,^136^ illustrating how environmental stress during sensitive developmental windows can induce long-lasting molecular and functional changes in PVIs.^135^ Nevertheless, because these findings were obtained in a combined neurodevelopmental and stress paradigm, they do not demonstrate an epigenetic mechanism induced by stress alone or occurring specifically within PVIs.

Evidence from PVI-specific Hdac2 deletion further demonstrates that epigenetic regulators can influence aggrecan expression, PNN organization, and PVI plasticit.^137^ However, this manipulation was not examined in a stress model. Thus, epigenetic regulation represents a plausible but still underdeveloped mechanism in stress-related PVI dysfunction. Cell-type-resolved methylomic, chromatin-accessibility, and transcriptional studies will be required to determine whether stress induces persistent epigenetic alterations directly within PVIs and whether these changes causally contribute to circuit and behavioral dysfunction.

## Differential PVI plasticity as a cellular correlation of resilience or vulnerability to stress

While reductions in PV immunoreactivity, PV-positive cell counts or density, and PVI function are frequently associated with vulnerability to stress-induced behavioral abnormalities, accumulating evidence indicates that these cells may also actively contribute to mechanisms of stress resilience. For instance, rats vulnerable to chronic stress exhibit impairments in GABAergic neurotransmission and a reduced number of PV-positive cell in the infralimbic PFC, whereas resilient animals show no such alterations.^138^ Furthermore, exposure to both escapable and inescapable footshock stress increases general neuronal activation in the medial PFC. However, only escapable stress increases *c-Fos* expression in PV/PNN-positive co-labeling cells in the infralimbic PFC and amygdala, suggesting a role for these cells in adaptive coping and resilience.^139^ In contrast to these changes observed in prefrontal regions, the number of PV-positive cell counts in the HIP does not appear to differ among controls, susceptible, and resilient rats in certain chronic stress models.^140^

Importantly, the metabolic, inflammatory, and epigenetic mechanisms described above may not only drive PVI dysfunction but also determine whether stress exposure leads to vulnerability or resilience. In addition, vulnerability and resilience mechanisms involving PVIs may differ between sexes. Reduction in PV-positive cell counts caused by adolescent stress were more pronounced in males,^36^^,^^70^^,^^101^^,^^141^ whereas females show greater individual variability in stress-induced alterations and age-dependent susceptibility.^142^^,^^143^ These sex differences may arise from interactions between hormonal fluctuations and genetic differences.^144^ PVIs express estrogen receptor β (ER-β) in both sexes, and in cortical regions over 95% of ER-β-positive cells correspond to PVIs.^143^^,^^145^ In females, puberty further amplifies PVI sensitivity to hormonal modulation, as estrogens drive remodeling of PNNs and regulate BDNF/TrkB signaling required for proper PVI maturation.^146^^,^^147^ Prepubertal ovariectomy attenuated adult UCMS-induced anxiety-like behavior. However, neither prepubertal ovariectomy, estradiol supplementation, nor UCMS altered overall PV expression in the prelimbic PFC. Instead, surgery, estradiol treatment, and stress showed interactive effects on PV/FosB colocalization, although post-hoc comparisons did not identify a robust stress-dependent effect.^148^ These findings suggest that estrogen may confer a developmental advantage to female PVIs, potentially shaping resilience to stress and contributing to sex-specific trajectories of psychiatric risk.^149^^,^^150^ Overall, current evidence suggests that sex differences in stress-related PVI plasticity primarily involve developmental timing, hormonal modulation, and the mechanisms recruited by stress. Although adolescent males more consistently show reductions in PVI/PNN-related measures, females display greater age-dependent variability, and whether these patterns translate into consistent sex-specific resilience remains unresolved.

Manipulation of PVI activity further supports their role in regulating stress-related behavioral outcomes. Chemogenetic inhibition of medial PFC PVIs induces helplessness behavior^69^ and can mimic behavioral alterations associated with juvenile social isolation, including abnormal aggression and impaired social preference.^151^ Conversely, enhancing PVI activity prevents stress-induced circuit dysfunction. For instance, chemogenetic activation of PVIs in the barrel cortex during restraint stress prevented excessive dendritic spine elimination in layer 5 pyramidal neurons in this region and mitigated recognition memory deficits.^152^ Similarly, selective activation of PVIs reduced dendritic spine loss during prolonged restraint stress exposure, whereas inhibition of these interneurons abolished the protective effects of ketamine on dendritic spine stability.

Recent studies have also revealed circuit-level mechanisms linking PVIs to stress resilience. Investigations into neural circuits underlying vulnerability and resilience to social defeat stress revealed a role for PVIs in the primary auditory cortex. In resilient mice, early aggressive encounters transiently hyperpolarized glutamatergic neurons in the medial geniculate body that project to the auditory cortex. This transient window of neural plasticity promotes the formation of new thalamocortical synapses onto cortical PVIs. This process is mediated by presynaptic signaling involving BDNF and its receptor TrkB during subsequent stress exposure, resulting in enhanced recruitment of PVIs and contributing to behavioral resilience.^153^

Consistent with findings in the PFC, hippocampal PVIs also play a key role in stress susceptibility. Chemogenetic inhibition of hippocampal PVIs induced social interaction deficits in mice exposed to subthreshold social defeat stress. Moreover, activation of PVIs in the ventral HIP following stress promoted susceptibility, whereas inhibition of these interneurons increased social interaction and sucrose preference in socially defeated mice.^118^ Transcriptomic analyses of hippocampal PVIs revealed that the mRNA encoding *Ahnak*, an endogenous regulator of L-type voltage-gated Ca^2+^ channels in PVIs, was significantly upregulated in susceptible mice compared to control or resilient animals. In contrast, resilient mice displayed a significant decrease in *Ahnak* expression compared to non-defeated control mice. Supporting a causal role, selective deletion of *Ahnak* in PVIs promotes resilience to social defeat stress.^118^

These findings highlight PVIs as key players of neural circuits underlying stress-related vulnerability and resilience. Rather than representing purely passive targets of stress-induced pathology, PVIs appear to actively shape adaptive circuit responses, suggesting that modulation of PVI plasticity may represent a promising strategy for promoting resilience to stress-related disorders.

## Mechanistic insights through therapeutic modulation of PVIs

Developing drugs capable of precisely modulating PVI function holds great promise. However, achieving this goal requires reliable translational biomarkers to measure target engagement and treatment efficacy in both preclinical models and patients. This section explores how different classes of pharmacological agents may modulate PVI function and/or restore it under stress-related conditions.

### Monoamines modulators

Monoaminergic systems are critically involved in stress responses from early-life to adulthood,^154–156^ and are implicated in the pathophysiology and treatment of mood and stress-related disorders.^157^ Given the sensitivity of PVIs to stress exposure, pharmacological modulation of monoamine signaling has also been considered as a potential strategy to restore PVI function and circuit stability following stress-induced alterations. Evidence regarding PVI/PNN indicates that the regulation of PVIs and their surrounding PNNs by monoaminergic antidepressants depends on factors such as brain region, treatment duration, stress history, and experimental protocol. Moreover, different classes of antidepressants appear to engage overlapping but different mechanisms.^158^^,^^159^

**Figure 2:**
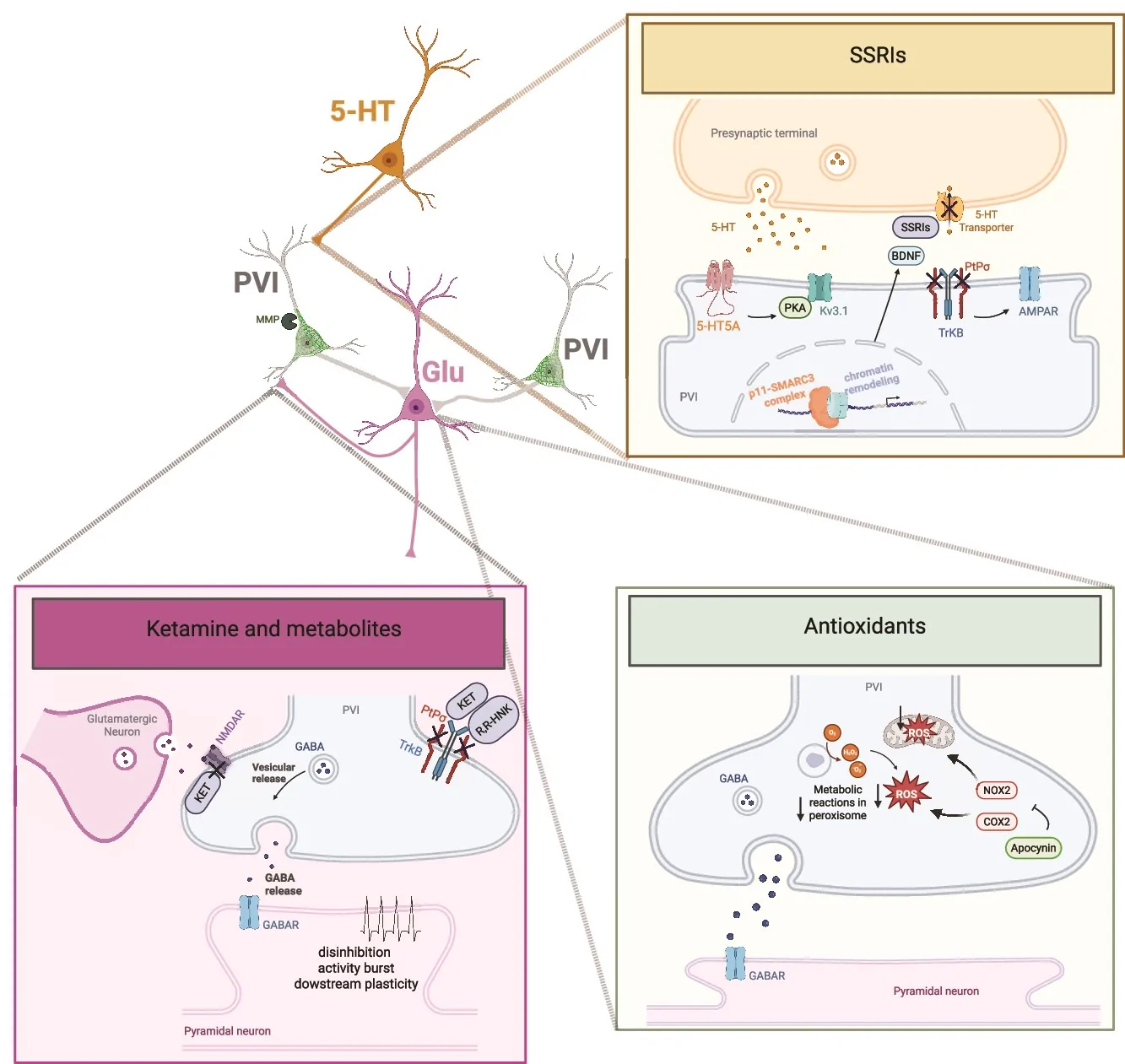
Pharmacological mechanisms modulating parvalbumin interneuron (PVI) stress-associated plasticity. The circuit illustrates the connections between serotonergic (5-HT), glutamatergic (Glu), and PVIs. Different classes of pharmacological interventions target these circuits via distinct pathways, restoring PVI function. The top-right panel shows that the SSRIs enhance 5-HT availability into PVIs. Activation of 5-HT5A receptors on PVIs triggers PKA-dependent phosphorylation of Kv3.1β channels, modulating interneuron firing rates. Fluoxetine promotes plasticity by disrupting the TRKB-PTPσ interaction, reopening critical period-like windows. Intracellularly, the p11–SMARCA3 complex promotes BDNF expression and AMPAR-dependent synaptic plasticity. The bottom-left panel shows the rapid-acting ketamine and its metabolites’ effects through the disinhibition of pyramidal neurons. Transient NMDA receptor blockade on PVIs reduces inhibitory tone, leading to an activity burst and downstream plasticity. Similar to SSRIs, ketamine and its metabolites disrupt TRKB-PTPσ complexes. In the bottom-right panel, the antioxidants show potential to restore redox homeostasis in PVIs. Interventions such as apocynin target the NOX2 and COX-2 pathways to inhibit oxidative stress. Reducing reactive oxygen species (ROS) protects PVIs from metabolic dysfunction, preserving network integrity and the excitatory/inhibitory balance.

One of the most consistently implicated mechanisms in antidepressant action involves modulation of the serotonin system. Drugs that enhance serotonin transmission, particularly selective serotonin reuptake inhibitors (SSRIs), represent frontline pharmacological treatments for depression and related disorders. In several animal studies, SSRIs have been shown to normalize stress- or disease-associated changes in PV immunoreactivity, PV-positive cell counts, or PV protein expression, depending on the model.^160–162^ In the HIP, behavioral responses to chronic SSRIs coincide with increased PVI activity. This effect appears to involve 5-HT5A receptors expressed in PVIs, which are initially functionally silent but become engaged after chronic SSRI exposure (Figure 2). Activation of these receptors initiates a signaling cascade through PKA pathway, leading to increased phosphorylation and inhibition of Kv3.1 channels, modulating the firing properties of PVIs. Studies suggest the effects of SSRIs on PVIs appear to be region- and layer-specific within the HIP.^163^^,^^164^ The antidepressant-induced plasticity may be related not only to changes in PV expression and function, but also to alterations in PNN integrity and, consequently, in the PNN and PVI relationship, indicating that both the interneuron population and its surrounding extracellular matrix can be affected and direct the changes by SSRIs.^165^^,^^166^ However, these effects could be context dependent. In unstressed animals (rodents and non-human primates), fluoxetine induces a dematuration process with reduced PV expression and decreased PNN coverage in hippocampal PVIs.^167^

Beyond their effects on PV expression and PNN remodeling, SSRIs also appear to regulate intracellular plasticity pathways within PVIs. Fluoxetine promotes phosphorylation of the TrkB receptor and reactivates critical period-like plasticity in the visual cortex. This mechanism involves the disruption of the interaction between TrkB and protein tyrosine phosphatase sigma (PTPσ), preventing TrkB dephosphorylation (Figure 2). By relieving this inhibitory interaction, fluoxetine enhances TrkB signaling in PVIs and reopens a window of heightened plasticity in adult visual circuits.^168^ Although this mechanism has been demonstrated primarily in the visual cortex, it will be interesting to corroborate whether similar signaling occurs in stress-sensitive regions. Another proposed mechanism involves the p11–SMARCA3 transcriptional complex. This complex mediates the antidepressant effects of chronic SSRIs by repressing Neurensin-2 expression in PVIs, thereby promoting BDNF expression and enhancing AMPA receptor-dependent synaptic plasticity in PVIs, implicating this pathway in the therapeutic actions of SSRIs^169^ (Figure 2).

Serotonin and norepinephrine reuptake inhibitors (SNRIs) antidepressants may also influence PVI function through the remodeling of the PNNs surrounding these cells. Findings indicate that in control mice, the chronic administration of venlafaxine has been associated with increased cortical levels of MMP-9, reduced PNN deposition, and enhanced power of gamma oscillations (*ex vivo*). In a model of chronic adolescent stress, venlafaxine treatment significantly increased the PNN/PV ratio and reversed associated impairments in cognition and sociability.^170^ Interestingly, the latency of the therapeutic response can be shortened when antidepressant treatment is combined with environmental enrichment, which further promotes PVI/PNN-related plasticity.^171^ Together, these findings suggest that MMP-9-mediated PNN remodeling may represent one mechanism by which SNRIs, such as venlafaxine, enhances PVI plasticity and function, thereby contributing to the recovery of gamma oscillatory activity and behavioral improvements.

Taken together, these findings indicate that monoaminergic antidepressants (especially SSRIs and SNRIs) may restore PVI function through multiple converging mechanisms. Beyond enhancing monoaminergic neurotransmission, these drugs promote PNN remodeling and engage intracellular plasticity-related pathways within PVIs. Such mechanisms may contribute to the normalization of network oscillations and behavioral outcomes following stress-induced circuit dysregulation.

### Ketamine and derivatives

As previously discussed, stress exposure is frequently associated with behavioral deficits, including depression-like states, accompanied by PVI disruption and functional alterations in inhibitory circuits. In this context, rapid-acting antidepressants such as ketamine and derivatives have attracted considerable attention for their ability to rapidly reverse both behavioral and cellular effects induced by stress. For example, esketamine has been shown to reverse stress-induced depressive-like behaviors and reductions in PVIs.^172^

One influential framework explaining ketamine’s antidepressant action is the disinhibition hypothesis. According to this model, ketamine exerts its effects by preferentially inhibiting GABAergic interneurons through the transient blockade of NMDA receptors, particularly those expressed in interneurons (Figure 2). This results in rapid disinhibition of excitatory pyramidal neurons, producing a transient *glutamate burst* that triggers downstream synaptic plasticity mechanisms in the medial PFC.^173^ Consistent with this model, ketamine has been shown to increase PVI activity following stress. It also counteracts the loss of PVI axonal boutons induced by repeated stress and significantly suppresses stress-induced spine elimination.^174^ The initial inhibition of PVIs may increase glutamatergic input onto these cells, leading to their subsequent activation. Supporting this interpretation, studies have reported an early decrease in GABAergic activity followed by a compensatory increase, occurring in parallel with a sustained enhancement of glutamatergic signaling.^175^ The disinhibition of pyramidal neurons leads to increased glutamate release, which in turn enhances activation of postsynaptic AMPA receptors on excitatory neurons^176^^,^^177^ (Figure 2). Importantly, the disinhibition hypothesis is not restricted to PVIs. Increasing evidence indicates that ketamine also modulates other inhibitory interneuron populations, particularly SST.^175^^,^^178^ Thus, the combined modulation of dendritic and perisomatic inhibition may contribute to the overall increase in pyramidal neuron excitability, re-establishing cortical output.

In addition, some studies report that PNNs are necessary for the antidepressant effects of ketamine, as enzymatic degradation of PNNs surrounding PVIs in the ventral HIP abolishes its sustained antidepressant action.^179^ On the other hand, work from Eero Castren’s group indicates that treatment with ketamine or its metabolite R,R-hydroxynorketamine (R,R-HNK) does not alter PNNs surrounding PVIs. These findings suggest that ketamine’s effects on PVIs may be mediated through intracellular signaling pathways rather than large-scale remodeling of extracellular matrix structures. Similar to fluoxetine, both ketamine and R,R-HNK can directly bind to TrkB receptor and disrupt its interaction with PTPσ, thereby promoting TrkB-dependent plasticity in PVIs^180^ (Figure 2). Some of these observations were made in the visual cortex, raising the possibility that the impact of ketamine on PVI/PNN dynamics may vary across brain regions. Finally, other rapid-acting antidepressants may share partially overlapping mechanisms with ketamine. For instance, scopolamine has been shown to produce rapid antidepressant effects that also involve inhibition of GABAergic interneurons, including PVIs.^178^ These mechanisms may ultimately contribute to the rapid normalization of cortical network dynamics and behavioral responses after stress-induced circuit dysfunction.

### Adjunctive therapies and potential new mechanisms

Pharmacological strategies targeting neuroinflammation, oxidative stress, and redox balance have also emerged as potential modulators of PVI vulnerability to stress. As discussed above, oxidative and inflammatory mechanisms contribute to stress-induced PVI dysfunction, making these pathways attractive targets for therapeutic intervention.

Anti-inflammatory treatments appear to prevent some of the effects of stress on PV expression. For instance, prophylactic inhibition of COX-2 during preadolescence prevented PV protein loss and improved working memory deficits induced by early-life stress in male rats^127^ (Figure 2). In females, COX-2 inhibition similarly prevented stress-induced reductions in PV protein levels and the development of depressive-like behaviors. Interestingly, COX-2 inhibition also decreased PV protein expression in non-stressed female rats,^181^ suggesting potential sex-specific or baseline-dependent effects of inflammatory signaling on PVIs.

Targeting oxidative stress pathways has also shown promise. Preventive treatment with the antioxidant N-acetylcysteine, administered either during or after adolescent stress exposure attenuated social and cognitive deficits, mitigated decreases in the number of PV- and PNN-positive cells in the ventral HIP and normalized the increased expression of the oxidative DNA damage marker 8-Oxo-dG in PV-positive cells,^182^ supporting a role for oxidative stress in PVI vulnerability. Consistent with this idea, NADPH oxidase 2 (NOX2), an enzyme responsible for generating superoxide, has been reported to be overexpressed in cortical regions of animals subjected to social isolation during adolescence. In these animals, increased NOX2 expression was accompanied by reductions in PV-positive cell density, as well as impairments in antioxidant defense systems and energy metabolism regulation. Treatment with apocynin, an antioxidant and NOX2 inhibitor, prevented both the behavioral and histopathological alterations induced by social isolation, including the loss of reductions in PV-immunoreactive cell density^183^ (Figure 2). Remarkably, apocynin treatment fully reversed behavioral alterations when initiated after four weeks of social isolation but was only partially effective when started after seven weeks, suggesting the existence of a time-sensitive window for therapeutic efficacy.^184^ Further support for the role of oxidative stress in PVI dysfunction comes from studies using a mouse model of NMDA receptor hypofunction in corticolimbic GABAergic interneurons (Ppp1r2-cre/fGluN1 KO mice). In this model, it was examined whether NOX-mediated oxidative stress contributes to the exacerbation of schizophrenia-like phenotypes induced by post-weaning social isolation. Chronic treatment with apocynin prevented the reduction in PV-positive cell density observed under these conditions.^185^

These findings highlight neuroinflammatory and oxidative stress pathways as contributors to stress-induced PVI dysfunction. Pharmacological strategies targeting these mechanisms, including anti-inflammatory agents and antioxidants, may therefore represent promising approaches to preserve PVI integrity and restore circuit function following stress exposure.

### Assessing human PVI function through translational biomarkers

The assessment of PVI function in psychiatric disorders and the development of targeted therapeutics depend on reliable translational biomarkers that can quantify PVI function and evaluate treatment efficacy across preclinical models and clinical populations.

Analysis of EEG revealed alterations in neural oscillatory activity across multiple psychiatric disorders and in preclinical studies. Although several classes of GABAergic interneurons contribute to the generation and modulation of cortical oscillations, evidence indicates that PVIs are important contributors to the synchronization of gamma-band activity.^186^ Nevertheless, gamma oscillations emerge from interactions multiple excitatory and inhibitory neuronal populations, and the relative contribution of PVIs depends on the cortical region, circuit architecture, and behavioral state. Furthermore, the interpretation of gamma activity can vary across resting-state, stimulus-evoked responses that are phase-locked to sensory events, and induced gamma activity that is not phase-locked and is thought to reflect higher-order perceptual and cognitive processes.^187^ Consistent with the importance of PVIs in such particular brain rhythm, abnormalities in gamma oscillations could possibly be a translational biomarker in psychiatry, as it can be measured using comparable paradigms in both humans and rodents.^186^^,^^188^ In psychiatry, this relationship has been most extensively characterized in schizophrenia, where reduced gamma-band synchrony, particularly in PFC networks during cognitive tasks has been associated with PVI dysfunction, including reductions in PV expression observed in human *postmortem* analysis. A deep revision of the topic can be found elsewhere.^189^

Another key biomarker that can be associated to EEG is the 40 Hz auditory steady-state response (ASSR). ASSR is elicited by periodic auditory tones near 40 Hz, a frequency that evokes the strongest response in humans and is also reliably observed in rodents. Because gamma oscillations, such as the 40 Hz ASSR, are primarily generated by the reciprocal interaction between pyramidal neurons and fast-spiking PVIs, they can serve as a functional biomarker for assessing PVI function and drugs that target them.^190^ Measuring the 40 Hz ASSR thus may provide a direct, non-invasive window into the health of this microcircuit, positioning it together with EEG as a promising translational biomarker that bridges human and animal models.

## Conclusion and future directions

PVIs are key regulators of cortical and hippocampal network dynamics, coordinating network synchronization and inhibitory control over pyramidal neurons. PVI dysfunction is increasingly recognized as a shared feature across several psychiatric disorders,^32^^,^^189^^,^^191^^,^^192^ and accumulating evidence indicates that PVIs are particularly vulnerable to stress, especially during sensitive developmental periods such as adolescence. Stress exposure may therefore act as a critical environmental factor that interacts with genetic susceptibility and developmental processes to destabilize PVI circuit function leading to the emergence of psychiatric disorders.

Multiple mechanism underlies stress-induced PVI dysfunction, including oxidative stress, neuroinflammatory signaling, alterations in neuromodulatory systems, and remodeling of PNNs. Collectively, the evidence supports a convergent model in which stress-related developmental, metabolic, and inflammatory alterations progressively impair PVI maturation and inhibitory function. This framework may help explain how stress destabilizes circuit activity and increases vulnerability to stress-related psychiatric phenotypes. These processes disrupt inhibitory circuit stability and impair network oscillations, ultimately contributing to behavioral and cognitive phenotypes relevant to stress-related psychiatric disorders. Targeting mechanisms that preserve or restore PVI function may offer new opportunities for the prevention and treatment of psychiatric disorders associated with stress exposure.

Despite significant advances, important gaps remain in our understanding of how stress affects PVIs. Future studies should aim to better characterize the diversity of PVI subtypes across brain regions, clarify the temporal dynamics of stress-induced alterations, and further investigate sex-specific mechanisms that may influence vulnerability and resilience. In addition, translating mechanistic insights from animal models to human studies remains a major challenge, highlighting the need for biomarkers and circuit-level measures that can capture PVI dysfunction in clinical populations.

## Data Availability

No new data was generated or analysed in support of this research.
